# Work, Health, and the Ongoing Pursuit of Health Equity

**DOI:** 10.3390/ijerph192114047

**Published:** 2022-10-28

**Authors:** Emily Q. Ahonen, Megan R. Winkler, Anjum Hajat

**Affiliations:** 1 Division of Occupational and Environmental Health, Department of Family and Preventive Medicine, University of Utah School of Medicine, Salt Lake City, UT 84108, USA; 2 Department of Behavioral, Social, and Health Education Sciences, Rollins School of Public Health, Emory University, Atlanta, GA 30322, USA; 3 Department of Epidemiology, University of Washington School of Public Health, Seattle, WA 98105, USA

The many facets of work, including employment relationships and attendant employment quality, the day-to-day conditions experienced in any given job, and the evolution of one’s working circumstances over time can support or detract from health, and combine in myriad ways to impact worker well-being. This not only occurs through exposure to hazards related to the conduct of job tasks, but is also influenced by the stability of work over time, the degree to which it allows one to develop and grow, the amount paid and the consistency of payment, whether workers receive sufficient resources and support to manage the demands of their work, how compatible the work is with the rest of life, and to what degree societies provide for economic support in times of non-work, to name a few factors. Put another way, people should be safe from harms to their health at work, but our societies are also set up such that, for many, much of the experience of the rest of life is dependent on—or at least closely intertwined with—work and what it brings us. Therefore, we must have work that is consistently safe from known hazards and also is an experience that can help to foster things that we need as humans, such as stability, inclusion, connection, respect, and a sense that we are well-rested, relaxed, vigorous, and flourishing.

Everyone needs these things, but they are not evenly distributed in populations, neither within political units nor across them, and evidence has accumulated showing that social stratification creates sub-populations of workers who systematically experience work-related factors that undermine health and well-being. When combined with other unevenly distributed resources and harms, those related to work contribute to the formation of health inequities visible at the level of populations. Making all work as health-supportive as possible and ensuring that it is distributed equitably is one way to move work toward a more just set of circumstances within and across societies.

The articles in this Special Issue come from researchers in six different countries and explore various aspects of work, health, and equity. The authors use study designs and logics derived from epidemiology, community-based (participatory) research, and the social sciences, employing qualitative, quantitative, and mixed-methods approaches to gather their data and undertake their analyses. As we considered the research, we categorized it into three general but not mutually exclusive content groupings, as follows:

1. Studies that highlight the ways in which work interacts with other social phenomena to influence health [1,2,3,4,5];
2. Studies that focus on the context in which work happens and emphasize the importance of that context in shaping work or health [6,7,8,9];
3. Studies that encourage us to think more expansively about work [10,11].

Most of the studies in this Special Issue fit the first category, and use common, socially relevant categories to examine patterns across groups. For instance, Gauffin and Dunlavy [1] focus on the self-employed in Sweden, a group that may be at risk for inequitable health outcomes because many forms of health support are tied to employment. Their results suggest that worker or employee statuses combine in complicated ways with income to shape health patterns in later life. While the study of income may not be a central focus of occupational health and safety researchers, the authors’ results suggest that it perhaps ought to be. Moreover, since research on the self-employed is rather limited, as are details of their circumstances in many data sets, the authors specify areas in whichresearchers should work toward a consensus about circumstances wherein whether someone is a worker or an employee is relevant to health. This may be particularly important as greater proportions of the population engage in self-employment. In another example, Peckham and colleagues [4] explore employment quality as a contributor to the longstanding phenomenon in which women generally report poorer health than men. Using counterfactual logic, they find that, if the distribution of employment quality across genders were more similar, women’s health would improve. Their results highlight the possibility of work-as-explanation and also work as a tool with which to improve known inequities in health status across social sub-groups. By showing the ways in which aspects of work contribute to health inequities across social groups, or by centering socially marginalized workers explicitly, all these contributions show us that work is part and parcel of the social creation of health inequity.

That is, work, like health, is socially created in specific contexts, and power dynamics shape that creation. Fujishiro, Ahonen, and Winkler [6] call upon us to center the influence of power resources in determining the quality of employment (and, therefore, its relative healthiness) in a given context. Locating power provides a framework that applies to all forms and types of work, and can, therefore, help to transcend the boundaries of employment circumstances, industry, or occupation. Interrogating the use of power as a common thread in work and health research can help us to identify patterns in the *drivers* of problems that take specific forms for specific groups of people, in specific places, that show up as health inequities. One aspect of patterning in the work/health relation is the legal framework within which work is conducted; work and health are addressed by legal frameworks and accepted practices at national or smaller units of aggregation. However, legal rights are tied to the societies in which they are developed, so they often have the same gaps and biases built into them that the society has, and these can create different circumstances regarding resources and protections for different socially defined groups of people. Vosko and colleagues [7] underscore the fact that context varies significantly among those within the same nation but under different legal constraints, demonstrating how the outsized power that agricultural employers have over migrant farmworkers, permitted by the legal context and reaching across national borders, limited the effectiveness of pandemic health protections for this group. 

Research on work and health also continues to evolve to better mirror work’s complexity as a factor influencing health. Researchers have integrated an eye for well-being as a relevant outcome, used research consensus to develop specific programs intended to promote health in the workplace, and contributed to guidelines and practices for workplaces that should make work better for health. Several of the contributions to this Special Issue suggest specific ways we might continue that progress by thinking more expansively about work. In particular, these papers call us to critically consider

(1) Where the boundaries of work begin and end;
(2) The need to expand and improve upon our occupational safety and health theories and concepts.

Tsui and colleagues [10] examine the concept of support in care work during the experience of client death, subjecting classic theories about the ties between work stressors and health to examination through a feminist lens. Looked at thusly, they show that a classic understanding of job stressors and support is insufficient to explain the experiences and well-being impacts for a large sector of the care workforce—and therefore insufficient to adequately protect and support this worker group, because a narrow lens presumes that work can be neatly segmented off from non-work life. In challenging the concept of a neat delineation between work and “non-work”, their research shows that, for care workers, work is a collective issue/problem/resource with health and well-being impacts that extend to their broader communities, as they seek support for the stressors they experience that their work does not adequately mitigate. In presenting a cohort profile for an ongoing study about economic engagement for urban residents, Richardson and colleagues [11] highlight employment as part of the complex path to economic stability. A major portion of their sample is informally or casually employed, which pushes us to expand our sense of which work is worthy of study, why people do the sorts of work they do, and how work could be better shaped to actually meet peoples’ needs and, therefore, better support their health. 

The authors in this Special Issue engaged with a range of issues and a variety of orientations in their research, making any number of categorizations of the included articles possible. In fact, many of these studies could have been placed into more than one of the categories we chose. Specifically, even when the authors’ focus was cross-group comparisons or documenting the experience of specific groups—such as those in our first category—they often also implied or stated the importance of other things, such as the context in which their population of focus navigated work. In studies in which the authors centered context, they sometimes did so as a way of thinking more expansively about what we think we know, or the domains that apply to the work–health–equity relationships. Finally, the studies that invited us to think more expansively about work did so through a close examination of how work is navigated by particular groups of people in concrete circumstances. 

Altogether, these approaches have the potential to advance the project of improving health inequities through studying and addressing work. Framing research questions within a specific area of knowledge, and then describing assumptions and exploring and testing the links between aspects of work and health for specific groups can build consensus about how work shapes health, what work is like for whom, and how that is relevant for population health equity. When we introduce context, we more easily recognize that the contours of work are not inevitable but, rather, modifiable, because we can see that the contours vary by place and time. We also gain clarity about where modification might be fruitful. When we question work’s parameters and engage our current theories for their limitations, we may see places where consensus ought to be limited, or we might shift that consensus in ways that open new directions to advance our knowledge. This latter goal may be especially important for researchers such as those in this Special Issue, who are focused on the work–health-equity relationship; work, as an institution, is one way that our societies shape health for people. As such, it is hard to imagine achieving population health equity without achieving equity in access to safe, healthy work that is supportive of broader human needs. We encourage researchers to be explicit in their research questions, designs, and dissemination regarding this goal. Achieving health equity will not happen by itself but, rather, will require collective effort to re-shape the conditions of society, including work. 
